# The Disinfecting Power of Antiseptics

**Published:** 1883-10

**Authors:** 


### SELECTIONS.

### The Disinfecting Power of Antiseptics.

In a paper laid before the French Academy, Dr. G. LeBon states that, as the result of his latest experiments on the antiseptic properties of the glyceroborates of sodium and calcium. He comes to the following conclusions:

1st. The disinfecting power of any antiseptic is in inverse ratio to the age of the putrefying material. A solution of chopped meat, six days old, will require a much smaller proportion of the disinfectant than when it has been kept, say, for a couple of months, when the amount required will be ten times greater.

2d. If we wish to measure the power of antiseptics by taking as a basis their disinfecting properties, when applied to chopped meat of normal strength (1—10), we shall find that the most powerful compounds are potassic permanganate, calcic hypochlorite, ferrous sulphate acidulated with acetic acid, carbolic acid, and potassic and sodic glyceroborate. For instance, in order to disinfect 10 cubic centimeters of the normal chopped meat solution mentioned above, we must take 500 cubic centimeters of a saturated solution of salicylic acid, 80 of a saturated solution of sodic glyceroborate, and several drops only of a 1 per cent solution of potassic permanganate.

3. There is no parallelism in the disinfecting action of an antiseptic and its action on microbes; potassic permanganate, for instance, although a most powerful disinfectant, exercises no power whatever on these organisms. On the other hand, alcohol, which, after a long time, checks their development, only acts as a very weak disinfectant,

4th. There is no parallelism between the power of an antiseptic in preventing putrefaction and its power of checking it, when once set up. Alcohol and carbolic acid, which are powerful preventives of putrefaction, act but feebly whan putrifaction has once commenced. Carbolic acid, therefore, when used in surgery, acts rather as a preservative than as an antiseptic.

5th. With the exception of a very small number of bodies, such as corrosive sublimate and other powerful poisons, the greater part of the antiseptics now in use, more especially carbolic acid, have been a very feeble action on bacteria. If we mix 20 cubic centimetres of the above mentioned normal solution of chopped meat with 50 or even 100 cubic centimetres of a saturated solution of carbolic acid, the larger bacteria are rendered motionless, while the smaller ones retain their vitality and powers of reproduction. The author possesses carbolized solutions four months old, still rich in bacteria. In fact, so far from looking on carbolic acid as a distroyer of bacteria, the author looks upon it as the best material for preserving their lives.

6th. The experiments hitherto made upon the cadaveric alkaloids have not solved the question as to whether the odorous volatile alkaloids evolved during putrefaction are poisonous or not, seeing that the products of putrefaction introduced into the body in these experiments contain bacteria, to which the resulting pois onous effects may or may not be attributed. After trying many experiments, the author simply introduced a number of frogs into a vessel, the bottom of which was covered with a layer of the normal meat solution already mentioned. When putrefaction first set in, although large quantities of sulphuretted hydrogen and other fetid products were evolved and the liquid swarmed with bacteria, the frogs did not seem to suffer in the slightest degree, although had an infinitesimal portion of the liquid been injected into an elephant, the animal would infallibly be poisoned. The same liquid kept for two months, and then, being harmless, when injected subcutaneously, killed the frogs in a few minutes when they were compelled to breathe its vapor. There is, therefore, no parallelism between the toxic properties of a putrefying liquid and of its emanations. On the contrary, they seem to be in inverse ratio—that is to say, the newer the liquid the more toxic it is, the older the liquid the more toxic are its exhalations.

7th. The small quantity of these volatile toxic alkaloids which is sufficient to kill animal life when breathed, shows them to rival nicotine, prussic acid, and other powerful poisons in their virulence.

8th. The author's experiments show why accidents have arisen when bodies have been exhumed after having been buried for a long time. The air of old cemeteries, although nearly free from microbes, is nevertheless extremely poisonous. The volatile products of putrefaction generated by microbes appear, therefore, to play an important part in contagious and infectious diseases.—*Manufacturer and Builder.*

